# Extended Reality Analgesia Evidence Reviews Often Lack Sufficient Intervention Detail

**DOI:** 10.1089/jmxr.2024.0043

**Published:** 2024-12-17

**Authors:** Susan Persky, Megan G. Jiao

**Affiliations:** 1 Social and Behavioral Research Branch, National Human Genome Research Institute, Bethesda, Maryland, USA.; 2 McGovern Medical School, University of Texas Health Science Center at Houston, Houston, Texas, USA.

**Keywords:** medical extended reality, evidence synthesis, research methods, content analysis, pain

## Abstract

**Background:**

Evidence synthesis projects such as systematic reviews and meta-analyses are defined by the focal research question addressed through assemblage and analysis of all relevant literature. In complex intervention domains such as medical extended reality (MXR), there are a plethora of intervention factors that could be included in research questions, which define study inclusion criteria and, in turn, shape the generalizability of results. This article quantifies how recently published evidence syntheses of MXR interventions for pain management characterize the primary studies they assess.

**Method:**

Inclusion criteria for analysis consisted of English-language scoping reviews, systematic reviews, and meta-analyses, published in 2021–2023, that evaluated MXR-based interventions for pain management in any setting. We employed quantitative content analysis to assess characterization of intervention features.

**Results:**

Of the 61 synthesis publications that met inclusion criteria, 29 (48%) included only minimal description of MXR intervention content, 14 (23%) included substantial content descriptions, and the remainder did not describe intervention content within synthesized studies. Hardware details were reported for 15 (25%) of publications in a minimal way, 28 (46%) in a substantial way, and not reported in 18 (30%) of syntheses. Among the 39 papers that included a meta-analysis, 10 (25%) explicitly evaluated the role of intervention features in intervention efficacy.

**Conclusion:**

Findings suggest considerable variability in the characterization of intervention elements (content and hardware), which can limit accurate conclusions about the generalizability of synthesis findings. Accordingly, we make recommendations to guide future evidence syntheses in the MXR domain.

## Introduction 

The evidence base for use of extended reality (XR) interventions in medical practice is rapidly accumulating across a variety of clinical areas. Evidence synthesis, such as, systematic review and meta-analysis, combines findings from individual trials to produce an overall picture of intervention effectiveness. As such, synthesis is a critical step toward determining which XR interventions are ready for translation to clinical care. The process of literature synthesis is bound by well-defined frameworks aimed at transparency and rigor.^1^ However, several aspects of evidence synthesis design are less prescribed and therefore are more variable between synthesis projects. In particular, synthesis scope and inclusion criteria are directly framed by authors’ research questions and necessarily take variable forms. In complex intervention domains like medical XR (MXR), there are a plethora of intervention facets (e.g., related to intervention characteristics, use case, setting) that could underlie research questions, define inclusion criteria, and in turn shape the eventual generalizability of synthesis results. This article quantifies the ways that recently published evidence syntheses of MXR interventions for pain management frame research questions and characterize the studies synthesized to address them. We focus, first, on the visibility of MXR intervention content (i.e., what users experience and perhaps interact with within the XR hardware). Content is a central property of MXR interventions; however, our anecdotal observations suggest that it is infrequently centered in evidence synthesis. Second, we address the way that XR hardware systems are defined and considered for inclusion in synthesis. Finally, we make recommendations for the consideration and characterization of intervention factors for future synthesis projects.

### Designing evidence synthesis

Evidence synthesis projects begin with a focal research question that is addressed through assemblage and analysis of all relevant literature. In the medical literature, frameworks such as “PICO”^2^ (participants, interventions, comparisons, outcomes) guide formulation of effective research questions with good specificity. Even with such frameworks, however, there are many nonstandardized decision points. For example, regarding MXR interventions for pain, PICO elements could be defined as follows: P(articipants) = adults, I(ntervention) = XR, C(omparisons) = any control group, and O(utcomes) = pain ratings, resulting in an exceedingly broad research question, such as: “Is use of XR for pain management in adults more effective than control?” This broad characterization leaves open whether and how intervention features such as hardware type, interactive elements, content, purported mechanism, and gamification are considered in the synthesis. PICO elements can, of course, be operationalized in a more specific way, resulting in a research question like: “Do immersive virtual reality (VR) distraction interventions result in lower doses of required pain medicine during pediatric wound care procedures vs. placebo control?” However, there is a limit to how specific such research questions can be. There are too many components comprising MXR interventions and too small a literature base to consider parsing all components into their smallest units. A synthesis of studies using “immersive VR underwater distraction environments using hand tracking for interactivity without gamification among adolescents receiving sutures in the emergency department,” for example, is not likely to be achievable at present.

As such, authors must make implicit and explicit choices about what to include in synthesis scope based on factors such as desired synthesis application and which intervention features are perceived to matter most. These choices have important consequences for the value of the synthesis to the field. Generally, methodologists posit that more variability across study components (i.e., broader research questions) translates to a less precise synthesis result and less generalizability to future interventions.^3^ Analyzing and understanding the effects of these components that cause variation (e.g., content type, intervention setting), either by narrowing synthesis scope to address specific intervention features or by including features as potential effect moderators, provides more precise information that is more directly applicable to future real-world intervention choices. It is therefore worth considering which features of interventions are already routinely integrated into MXR evidence synthesis and where recommendations for improvement of future syntheses might be helpful.

### Defining the MXR intervention

**FIG. 1.**
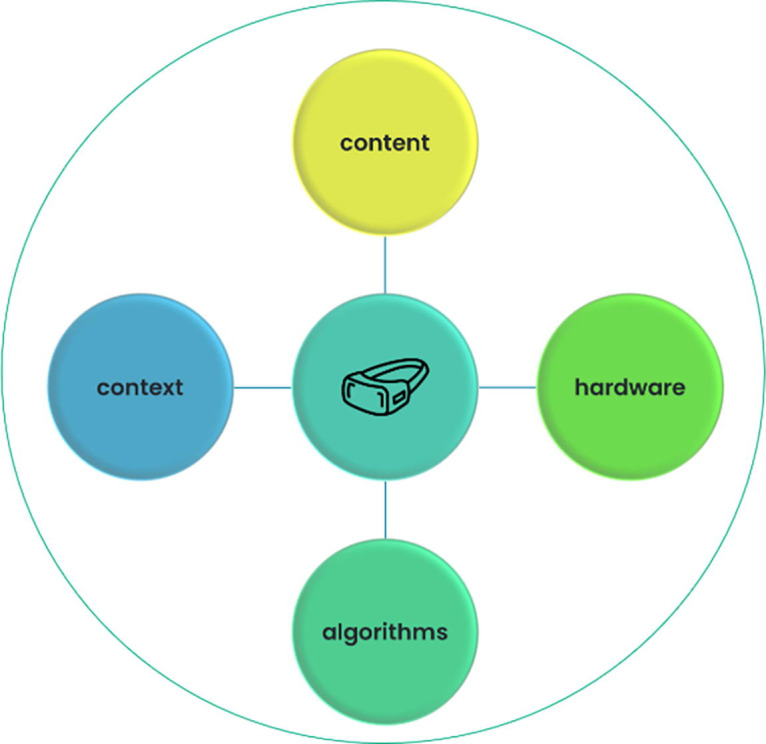
A holistic definition of an extended reality-based intervention.

Part of the challenge with designing more precise evidence syntheses is that there is no standard definition of an “MXR intervention.” Discussions of MXR applications often center upon use of a headset as the primary (or only) defining intervention feature. Media reports and academic paper titles pose questions such as, “Can VR effectively treat chronic pain?,” accompanied by photos of people wearing headsets with arms outstretched. This representation overemphasizes the importance of the headset as the underlying cause of efficacy in MXR interventions. Many XR researchers instead consider XR hardware similarly to a syringe or pill capsule that (1) delivers intervention material, and (2) can be designed to contribute to the efficacy of the intervention. XR hardware and software can synergistically make the whole more powerful, just as a capsule can deliver a drug more slowly or precisely. For example, ability to control XR systems with direct body movement, as enabled by hardware, makes physical engagement with digital game content more effective for physical rehabilitation therapy. Hardware and content are accompanied by other elements in interventions, including programmatic algorithms tying hardware, software, and user together (e.g., controlling the path and speed of digital targets in a therapeutic rehabilitation application based on user performance on prior trials), the broader delivery context (e.g., medical staff involvement, user training), and the synergistic influences of all four components. Taken all together, these elements might more appropriately define “the MXR intervention,” which could form the unit of analysis for evaluation and evidence synthesis (Fig. 1). In this way, a given MXR intervention “type” might be comprised of (1) content (e.g., a virtual game that requires visual object tracking and making hand contact with that object), (2) hardware (e.g., an untethered, standalone VR system with hand tracking), (3) algorithms (e.g., increasing difficulty based on performance), and (4) context (e.g., used in outpatient clinics with no adjunctive therapies). At a minimum, however, description of MXR interventions in evidence syntheses should encompass both hardware and software elements. Evaluations meeting these criteria will be better positioned to identify efficacious intervention elements and delineate aspects of interventions most amenable to alteration and optimization.

### The centrality of content

While XR hardware has relatively similar features across most product lines, content within an intervention can be incredibly heterogeneous, entailing applications that are custom-developed or commercial off-the-shelf. For this reason, characterization of XR content is perhaps most critical in evidence synthesis.

It is difficult to characterize how widely MXR intervention content varies within a clinical use case. However, there are currently many content approaches leveraged for MXR interventions, and at this stage, many published trials of MXR applications involve content that was created bespoke or adapted for a one-off trial. In the area of pain management, for example, MXR intervention content can include one or more of the following: 360-degree video environments, pain education, biofeedback sessions, cognitive-behavioral therapy instruction, immersive stories, and interactive games. While each of these content types addresses the same clinical outcome (i.e., pain), they operate differently and may have differential effectiveness within particular pain contexts. When evidence syntheses should keep such variations in content distinct versus merging them is not yet clear. However, synthesis projects that do not consider or review content as an element of MXR interventions lack the specificity needed to effectively support decisions about future clinical implementation. Indeed, the broad conclusion that “XR interventions work for pain management” without consideration of XR content could suggest that simply donning a headset devoid of any content will reduce patients’ pain. Thus, it is critical that content is adequately characterized when assessing the efficacy of a class of MXR interventions in synthesis projects.

### What is XR? Variability in hardware

XR hardware is also a vital topic to address, given its role in enabling and enhancing content delivery. Discussion of XR hardware is essentially a given in evidence syntheses of this nature. However, there is wide variation in how XR hardware is defined. As such, it is important that synthesis projects not take the term at face value but instead delineate the hardware and systems that operationalize “XR” in their analysis. This is the case for all forms of XR but can been seen readily in the case of VR. For example, the VR literature includes both “immersive” and “nonimmersive” varieties. Here, we define immersive VR as a system including hardware that envelops the senses and creates sensory separation from the physical space, most often operationalized by use of a VR headset. In contrast, nonimmersive VR is defined as a system that administers three-dimensional content using a digital display that does not or only partially envelopes the senses, such as a tablet. While there have been many conceptual framings of VR over the years, more modern conceptualizations have focused on the headset (and thus, immersion) as a key element. Although there are no data on this point, many readers may assume that all studies in a review of VR applications would involve a headset or other immersive hardware, given popular depiction of VR as nearly synonymous with headset displays. From this vantage, some nonimmersive systems might be better positioned as a comparator or control group in a trial as opposed to representing VR. At the end of the day, however, the purpose of the review and the ways in which authors wish it to generalize will dictate whether or not nonimmersive VR applications should be included, and it is critical that this be clearly communicated and discussed as part of the larger analysis. The same is true for other XR system types, such as augmented and mixed reality.

### The current study

This study surveyed recent published evidence synthesis reports in pain treatment and management to assess how the MXR research community currently defines, categorizes, and assesses research studies vis-a-vis the inclusion and explicit characterization of intervention content and hardware configuration. Through quantitative content analysis, we bring data to the question of how the evidence syntheses used to determine efficacy and inform clinical implementation explicitly consider two core elements of MXR interventions: content and hardware.

We conducted this analysis in the domain of pain management because it is one of the most common and fruitful areas of MXR clinical research to date.^4^ It is also an area wherein intervention content varies quite widely. MXR interventions in pain are also a priority area for evidence synthesis given the already large and varied evidence base. Nuanced parsing of this evidence base could more effectively aid in preparations for MXR pain interventions to emerge as a mainstream clinical tool and inform evidence synthesis for other MXR clinical applications as well.

## Method

**FIG. 2.**
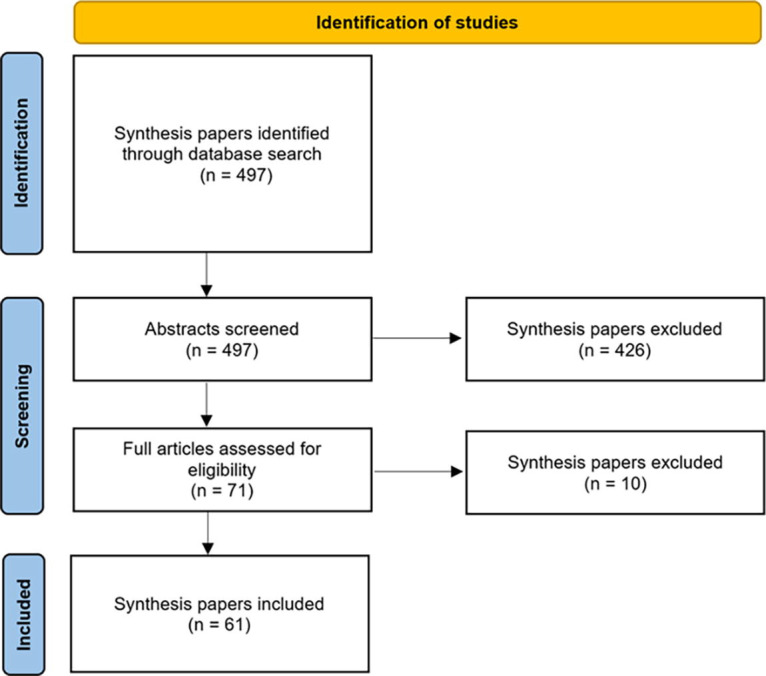
Paper search and inclusion strategy.

Inclusion criteria for analysis consisted of English-language scoping reviews, systematic reviews, and meta-analyses published in 2021–2023 that evaluated XR-based interventions for pain management in adult and pediatric populations across any setting (e.g., inpatient, outpatient). We chose this time frame to capture only recent published reviews. Reviews assessing XR intervention influence on multiple outcomes were required to include pain-oriented endpoints among the primary three outcomes assessed. To be eligible for inclusion, reviews must have included studies evaluating VR-based interventions but could also include other interventions along the XR continuum. Predetermined search terms included the following: (((review) OR (systematic) OR (meta-analysis) OR (scoping) OR (comprehensive)) AND ((virtual reality) OR (VR) OR (virtual world) OR (virtual environment) OR (mixed reality) OR (extended reality) OR (immersive) OR (immersi*)) AND ((pain) OR (analgesia) OR (analges*) OR (anesthesia) OR (anesthes*) OR (nocicep*) OR (distraction) OR (distract*))). These were entered into Pubmed and limited to the prespecified time frame, resulting in 497 total identified records. After screening for inclusion and eligibility criteria (see Fig. 2),^61^ papers were included in this analysis.

The codebook (Table 1) used to evaluate synthesis content and reporting approaches was developed based on inductive and deductive approaches with the primary aim of assessing the presence and quality of XR content and hardware descriptions within each literature synthesis publication. Coding items assessed the presence and nature of content descriptions included for the individual empirical research studies that comprised the reviewed material within the synthesis. For example, when coding for the presence of content, we examined whether and how extensively a given published synthesis provided details about the content experienced in each individual study within the review set.

**Table 1.** Codebook for Content Analysis

Coding item	Definition	Response options
Synthesis type	Type of synthesis performed on the included data, gleaned from authors’ description of the analysis in paper title, abstract, and/or body. More than one can be selected.	Scoping review Systematic review Meta-analysis
Number of studies	Full number of studies included in synthesis, whether or not a given study focuses on XR (e.g., studies that included non-XR digital health interventions in addition to XR were counted).	Numeric
Pain type	Type of pain addressed by the evidence synthesis. Gleaned from authors’ description in the paper title, abstract, and/or body. More than one can be selected. To distinguish from chronic pain, rehabilitation-related pain was defined narrowly as pain experienced during recovery from a procedure or injury. For example, this would include rehabilitation following a knee replacement but not rehabilitation for chronic back pain.	Chronic Acute Rehabilitation-related
Population included (if specified) and therapeutic target	What population(s) were included in the evidence synthesis, which entailed adults, children, or both, with the addition of specific characteristics, as appropriate (e.g., children with cancer). Some studies specified inclusion criteria that did not align with the ultimate populations included; in these cases, the actual populations included were used. Some studies included women, which were labelled accordingly in ^Table 2^, but these were grouped under studies including adults for quantification in the main text. Therapeutic target refers to the main clinical outcome(s) addressed, derived directly from the methods.	Population included: Adults (+/− additional characteristics) Children (+/− additional characteristics) Adults and children (+/− additional characteristics) Women Therapeutic target: Variable
Content description (1) In title/abstract (2) In paper body	Content description details what participants “do” and engage with in the XR setting (e.g., “a racing game”) in each study included in synthesis. Excludes solely mechanistic/functional descriptions (e.g., “exposure”). For content in the title or abstract, only presence was evaluated with “yes” or “no.” For content in the paper body (e.g., results, discussion, tables, and figures), extent of content was evaluated. “Substantial” required specifics for at least 2 of the following for at least half of the study entries in the review: (1) description of the visual environment, (2) interactive nature of stimuli, (3) additional audio or other sensory stimuli, and (4) specific product names. “Minimal” is less than substantial (but more than none) in the amount of information provided about each study or the number of studies described with “substantial”-level granularity.	Title/abstract: Yes No Paper body: None Minimal Substantial
Hardware specification	Hardware specification details what type of equipment was used to experience therapeutic content (e.g., an Oculus Quest headset; PC VR headset) in each study included in synthesis. “Substantial” should provide specific hardware specs or product names for at least half of included studies. “Minimal” is less than substantial (but more than none) in terms of the amount of information provided about each study vis a vis equipment or the number of studies described with “substantial”-level granularity.	None Minimal Substantial
Type of VR hardware and presence of other XR	Types of XR hardware (VR, augmented reality (AR), etc.) in each study included in synthesis. Based on types of hardware that were actually included after reading individual study descriptions where possible rather than a blanket inclusion description. VR hardware was classified further, whereas inclusion of other XR hardware (e.g., AR, mixed reality) was mentioned more generally.	Immersive VR Immersive and nonimmersive VR Nonimmersive VR Unspecified VR +/− other XR hardware
Key XR concepts discussed	Whether and, if so, which intervention elements were discussed as a topic in the evidence synthesis in the context of the included studies. Thus, abstract discussions that were not grounded in included studies were excluded. This includes reporting of frequency of these factors among included studies.	Immersion Interactivity Additional characterization
Intervention moderators evaluated	Whether and, if so, what moderators related to study interventions were used in reviews with a meta-analysis to segment analysis such that the synthesis reports differences in efficacy based on a given element. A study can have more than one moderator evaluated. “No” indicates that either moderators were present but were not related to the intervention (e.g., clinical outcomes, study design, etc.) or the meta-analysis did not include moderators. “N/A” indicates that there was no meta-analysis present, so moderators could not be determined.	Yes Content Interactivity Immersion Adjunctive Age group Other No N/A

VR, virtual reality; XR, extended reality.

The codebook was employed by two trained coders who reached benchmark interrater reliability statistics of kappa = 0.60 or greater for all subjective codes on 30 percent of the data set. Coders met to engage in reconciliation and arrive at a final set of codes. The remaining entries were coded by a single trained coder.

## Results

**FIG. 3.**
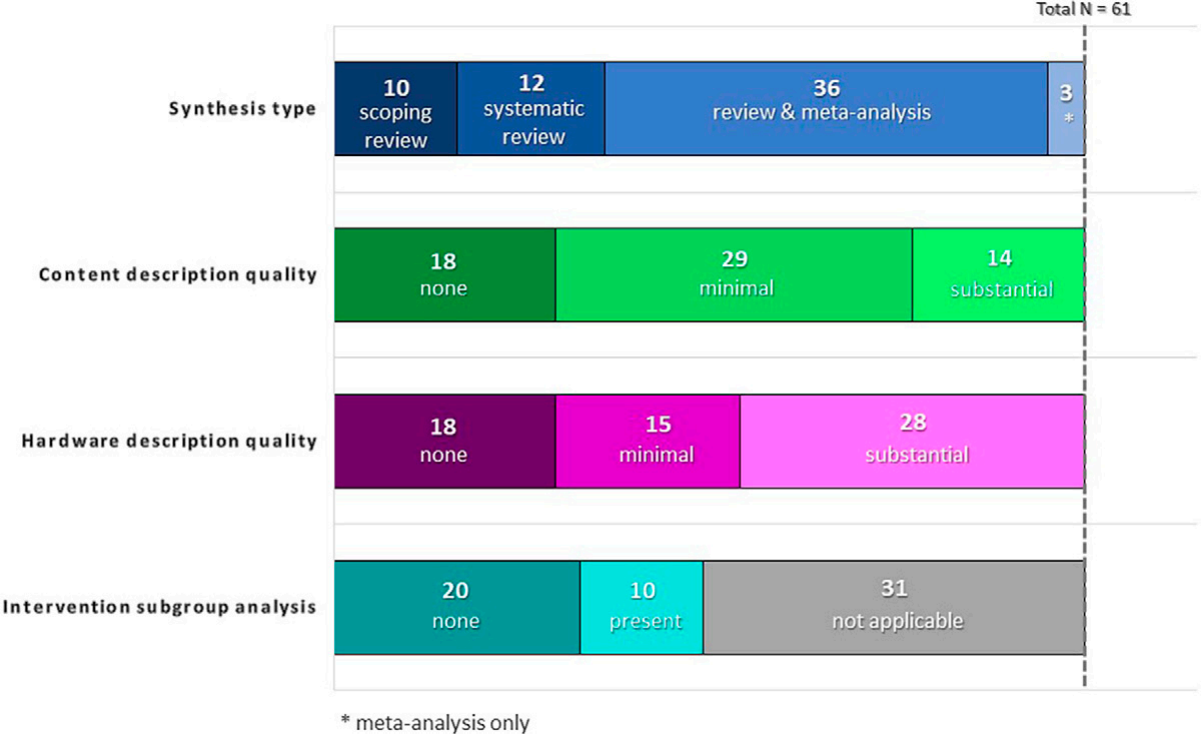
Feature frequency in sample of synthesis papers.

Of the 61 synthesis publications that met inclusion criteria, there were 10 scoping reviews, 12 purely systematic reviews, 36 combination systematic reviews and meta-analyses, and 3 meta-analysis-only (i.e., without narrative review; see Table 2, Fig. 3) reports. The number of studies synthesized in each review ranged from 4 to 122, with an average of 16.4 studies and median of 10 studies per synthesis. Twenty-six (43%) reviews included only interventions targeting adults, 19 (31%) included only ones targeting children, and 8 (13%) included both. Eight (13%) did not specify the populations included in terms of age group. Twenty-four (39%) reviews focused on management of acute pain (e.g., wound care, procedure-related pain), while 26 (43%) focused on addressing chronic pain (e.g., phantom limb pain, musculoskeletal pain), and 5 (8%) included both acute and chronic pain. Additionally, six (10%) reviews assessed pain in the context of rehabilitation, one of which targeted both acute pain and rehabilitation-related pain.

**Table 2.** Review Characteristics

Authors and year of publication	Synthesis type	Number of studies	Population included (if specified); therapeutic target	Pain type
Arcos-Holzinger et al. (2023)^5^	Scoping	7	Adults; complex regional pain syndrome	Chronic
Austin (2022)^6^	Scoping	44	Adults; primary and secondary chronic pain conditions	Chronic
Austin & Siddall (2021)^7^	Scoping	9	Adults; neuropathic pain in spinal cord injuries	Chronic
Baker et al. (2022)^8^	Scoping	70	Adults and children; acute and chronic pain	Acute and chronic
Baradwan et al. (2022)^9^	Systematic and meta-analysis	8	Women; labor pain	Acute
Bilika et al. (2023)^10^	Scoping	9	Adults; chronic musculoskeletal pain	Chronic
Blasco et al. (2021)^11^	Systematic	6	Total knee replacement rehabilitation	Rehabilitation
Bordeleau et al. (2022)^12^	Systematic and meta-analysis	24	Adults; back pain rehabilitation	Chronic
Boyce et al. (2023)^13^	Systematic	16	Adults; patient experience during awake invasive procedures	Acute
Brea-Gomez et al. (2021)^14^	Systematic and meta-analysis	14	Adults; chronic lower back pain	Chronic
Burrai et al. (2023)^15^	Systematic and meta-analysis	8	Adults with cancer receiving chemotherapy; anxiety, fatigue, and pain	Chronic
Cheng et al. (2022)^16^	Systematic and meta-analysis	6	Children with cancer; pain, anxiety, and fear	Chronic
Choi et al. (2023)^17^	Systematic and meta-analysis	11	Adults; low back pain (musculoskeletal pain, neuralgia, visceral pain, mechanical pain, nociceptive pain)	Chronic
Cohen et al. (2023)^18^	Systematic and meta-analysis	4	Pain and anxiety for office hysteroscopy	Acute
Comparcini et al. (2023)^19^	Systematic	13	Children with cancer; pain and anxiety while undergoing painful and/or anxiety-inducing medical procedures/cancer treatments	Chronic
Cortés-Pérez et al. (2021)^20^	Systematic and meta-analysis	11	Women; fibromyalgia	Chronic
Czech et al. (2021)^21^	Systematic and meta-analysis	7	Children; pain, fear, and anxiety during needle-related medical procedures	Acute
Czech et al. (2022)^22^	Systematic and meta-analysis	17	Adults and children; wound care and rehabilitation after burns	Acute and rehabilitation
Czech et al. (2023)^23^	Systematic and meta-analysis	9	Children with cancer; physical functions, fear, quality of life (pain, anxiety) while undergoing chemotherapy	Chronic
Cunningham et al. (2021)^24^	Systematic	4	Children; pain and anxiety while undergoing dental examination or treatment	Acute
de Jesus Catalã et al. (2022)^25^	Systematic	4	Children; burn pain	Acute
Dreesmann et al. (2022)^26^	Systematic	23	Adults; acute pain	Acute
Eldaly et al. (2022)^27^	Systematic	9	Adults; phantom limb pain	Chronic
Gao et al. (2022)^28^	Systematic and meta-analysis	27	Children; pain, anxiety, and fear of needle-related procedures	Acute
Garrido-Ardila et al. (2022)^29^	Systematic	10	Adults and children with burns; pain and range of joint movement	Rehabilitation
Gazendam et al. (2022)^30^	Systematic and meta-analysis	9	Adults; rehabilitation following total knee arthroplasty	Rehabilitation
Goudman et al. (2022)^31^	Systematic and meta-analysis	41	Adults and children; chronic pain (primary and secondary)	Chronic
Grassini (2022)^32^	Systematic and meta-analysis	9	Chronic neck/low back pain	Chronic
Guo et al. (2023)^33^	Systematic and meta-analysis	8	Adults and children; neck pain	Acute and chronic
Hali et al. (2023)^34^	Systematic	15	Adults; phantom limb pain	Chronic
He et al. (2022)^35^	Systematic and meta-analysis	13	Adults; pain in wound care	Acute
Huang et al. (2022)^36^	Systematic and meta-analysis	31	Adults and children; pain management	Acute and chronic
Jenabi et al. (2023)^37^	Systematic and meta-analysis	9	Children; pain during intravenous injection	Acute
Kantha et al. (2023)^38^	Systematic and meta-analysis	9	Adults; pain in chronic musculoskeletal disorders	Chronic
Kodavi et al. (2023)^39^	Systematic and meta-analysis	10	Adults; pain and anxiety during the periprocedural period	Acute
Kumar et al. (2023)^40^	Systematic and meta-analysis	7	Adults; chronic low back ache	Chronic
Li et al. (2022)^41^	Meta-analysis	4	Children; procedural pain from wound manipulation	Acute
Lier et al. (2023)^42^	Systematic and meta-analysis	122	Pain management	Acute and chronic
Lluesma-Vidal et al. (2022)^43^	Systematic and meta-analysis	21	Children; pain and fear during procedures involving needles	Acute
Martinez-Bernal et al. (2023)^44^	Scoping	27	Periprocedural pain and anxiety in oral cavity procedures	Acute
Matthie et al. (2022)^44^	Systematic	46	Adults and children; chronic pain (primary and secondary)	Chronic
Mazaheri et al. (2023)^45^	Systematic and meta-analysis	14	Adults; pain during wound care	Acute
McCahill et al. (2021)^46^	Scoping	4	Children; pain and anxiety during procedures in the emergency department	Acute
Merino-Lobato et al. (2023)^47^	Systematic and meta-analysis	21	Children; pain and anxiety during venipuncture	Acute
Nagpal et al. (2022)^48^	Scoping	13	Chronic low back pain	Chronic
Norouzkhani et al. (2022)^49^	Systematic and meta-analysis	30	Adults and children with burns; pain during wound care	Acute
O’Connor et al. (2022)^50^	Scoping	7	Adults; chronic pain	Chronic
Ozturk et al. (2023)^51^	Systematic and meta-analysis	7	Children with cancer receiving treatment; anxiety and pain	Acute and chronic
Peng et al. (2021)^52^	Systematic and meta-analysis	8	Adults; rehabilitation following total knee arthroplasty	Rehabilitation
Rajendram et al. (2022)^53^	Systematic and meta-analysis	15	Phantom limb pain	Chronic
Saliba et al. (2022)^54^	Systematic and meta-analysis	10	Children; pain, fear, and anxiety while undergoing vascular access procedures	Acute
Savaş et al. (2023)^55^	Systematic and meta-analysis	6	Children; pain during burn dressing	Acute
Singleton et al. (2023)^56^	Scoping	5	Children; chronic pain or pruritis	Chronic
Smith et al. (2022)^57^	Systematic and meta-analysis	10	Children with burns; pain and anxiety while undergoing burn wound care	Acute
Su et al. (2023)^58^	Systematic and meta-analysis	14	Adults; rehabilitation in total knee arthroplasty	Rehabilitation
Tas et al. (2022)^59^	Systematic and meta-analysis	26	Children; pain and anxiety during medical procedures	Acute
Vassantachart et al. (2022)^60^	Systematic	20	Phantom limb pain	Chronic
Vitagliano et al. (2023)^61^	Meta-analysis	5	Women; pain during outpatient hysteroscopy	Acute
Wang et al. (2022)^62^	Meta-analysis	10	Children; pain, fear, and anxiety during needle-related procedures	Acute
Wong et al. (2022)^63^	Systematic	17	Adults; chronic pain, anxiety, depression, mood	Chronic
Ye et al. (2023)^14,64^	Systematic and meta-analysis	5	Adults; chronic nonspecific neck pain	Chronic

### Inclusion of content and quality of description

Of the 61 included synthesis papers, two (3%) described MXR content generally in the abstract in addition to the paper body, while 43 (70%) mentioned content in the body of the paper, either in the text, tables, or figures (Table 3); 18 (30%) did not describe intervention content in reviewed studies at all. Out of all reviews, 29 (48%) included only minimal content descriptions (e.g., “therapeutic games on Xbox”^29^), while 14 (23%) included substantial content descriptions (e.g., “a game called Virtual River cruise. The game involved guiding a boat to shore, with snow sculptures in the middle of the road where the participants could guide the boat by shaking their head and receiving the sculptures”^49^). Four papers (7%) included content descriptions only in supplemental or appendix tables, all of which were minimal in their description.

**Table 3.** Presence of Content and Characterization in Reviews

Authors and year of publication	Content in title or abstract	Content in paper body	Hardware specification	Type of VR and presence of other XR	Intervention moderators evaluated	Key concepts discussed
Arcos-Holzinger et al. (2023)^5^	No	Substantial	Substantial	Immersive VR only	N/A	Additional Characterization
Austin (2022)^6^	No	Minimal	Minimal	Immersive VR, nonimmersive VR	N/A	Immersivity
Austin & Siddall (2021)^7^	No	Minimal	Substantial	Immersive VR, nonimmersive VR	N/A	Immersivity, Interactivity
Baker et al. (2022)^8^	No	None	Substantial	Immersive VR only	N/A	Interactivity, Additional Characterization
Baradwan et al. (2022)^9^	No	Minimal	None	Unspecified VR	No	None
Bilika et al. (2023)^10^	No	Minimal	Substantial	Immersive VR only	N/A	Immersivity
Blasco et al. (2021)^11^	No	None	Substantial	Nonimmersive VR	N/A	None
Bordeleau et al. (2022)^12^	Yes	Substantial	Substantial	Immersive VR, nonimmersive VR	No	Immersivity, Additional Characterization
Boyce et al. (2023)^13^	No	Substantial	Substantial	Immersive VR only	No	Immersivity, Additional Characterization
Brea-Gomez et al. (2021)^14^	No	Substantial	Substantial	Immersive VR, nonimmersive VR, AR	Alone vs. adjunctive Hardware type and content type (combined)	Additional Characterization
Burrai et al. (2023)^15^	No	Substantial	Substantial	Immersive VR only	No	None
Cheng et al. (2022)^16^	No	Minimal	None	Unspecified VR	No	None
Choi et al. (2023)^17^	No	None	None	Immersive VR, nonimmersive VR	No	Immersivity
Cohen et al. (2023)^18^	No	None	Substantial	Immersive VR only	No	None
Comparcini et al. (2023)^19^	No	Substantial	Substantial	Immersive VR, nonimmersive VR	N/A	Immersivity
Cortés-Pérez et al. (2021)^20^	No	None	None	Immersive VR, nonimmersive VR	Alone vs. adjunctive	Immersivity
Czech et al. (2021)^21^	No	Minimal	Substantial	Immersive VR only	No	None
Czech et al. (2022)^22^	No	Minimal	Minimal	Immersive VR, nonimmersive VR	No	Immersivity
Czech et al. (2023)^23^	No	Minimal	Substantial	Immersive VR, nonimmersive VR	No	Immersivity
Cunningham et al. (2021)^24^	No	None	Minimal	Immersive VR only	N/A	None
de Jesus Catalã et al. (2022)^25^	No	Minimal	Minimal	Nonimmersive VR	N/A	None
Dreesmann et al. (2022)^26^	No	Minimal	Minimal	Immersive VR only	N/A	Immersivity, Interactivity
Eldaly et al. (2022)^27^	No	Substantial	None	Unspecified VR, AR	N/A	None
Gao et al. (2022)^28^	No	None	None	Unspecified VR	No	None
Garrido-Ardila et al. (2022)^29^	No	Minimal	Minimal	Immersive VR, nonimmersive VR	N/A	None
Gazendam et al. (2022)^30^	No	Minimal	Minimal	Immersive VR, nonimmersive VR	No	None
Goudman et al. (2022)^31^	No	Substantial	Substantial	Immersive VR, nonimmersive VR	Immersive vs. nonimmersive	Immersivity, Additional Characterization
Grassini (2022)^32^	No	Minimal	Minimal	Immersive VR, nonimmersive VR	No	None
Guo et al. (2023)^33^	No	Substantial	None	Immersive VR only	Alone vs. adjunctive	None
Hali et al. (2023)^34^	No	None	None	Immersive VR only	N/A	Interactivity
He et al. (2022)^35^	No	None	Substantial	Immersive VR, nonimmersive VR	No	None
Huang et al. (2022)^36^	No	Minimal	Substantial	Immersive VR, nonimmersive VR	Targeted age group	None
Jenabi et al. (2023)^37^	No	Minimal	Minimal	Immersive VR only	No	None
Kantha et al. (2023)^38^	No	Minimal	Substantial	Immersive VR, nonimmersive VR	Immersive vs. nonimmersive	Immersivity, Additional Characterization
Kodavi et al. (2023)^39^	No	None	None	Immersive VR, nonimmersive VR	No	Immersivity
Kumar et al. (2023)^40^	No	None	Substantial	Immersive VR only	No	None
Li et al. (2022)^41^	No	Minimal	Minimal	Unspecified VR	No	None
Lier et al. (2023)^42^	Yes	Minimal	None	Immersive VR, nonimmersive VR	Content type Interactivity level Targeted age group Gamification	Immersivity, Interactivity, Additional Characterization
Lluesma-Vidal et al. (2022)^43^	No	Minimal	Substantial	Immersive VR only	No	None
Martinez-Bernal et al. (2023)^44^	No	Minimal	Substantial	Immersive VR, nonimmersive VR	N/A	Immersivity, Interactivity, Additional Characterization
Matthie et al. (2022)	No	Minimal	Substantial	Immersive VR, nonimmersive VR, AR + MR	N/A	Immersivity, Interactivity, Additional Characterization
Mazaheri et al. (2023)^45^	No	Substantial	Substantial	Immersive VR only	No	Interactivity, Additional Characterization
McCahill et al. (2021)^46^	No	None	None	Unspecified VR	N/A	None
Merino-Lobato et al. (2023)^47^	No	None	None	Unspecified VR	No	None
Nagpal et al. (2022)^48^	No	None	None	Unspecified VR	N/A	None
Norouzkhani et al. (2022)^49^	No	Substantial	Minimal	Immersive VR, nonimmersive VR, AR	Immersive vs. nonimmersive Targeted age group	Immersivity
O’Connor et al. (2022)^50^	No	Minimal	Substantial	Immersive VR, nonimmersive VR	N/A	Additional Characterization
Ozturk et al. (2023)^51^	No	Minimal	Substantial	Unspecified VR	Targeted age group	None
Peng et al. (2021)^52^	No	Minimal	Substantial	Immersive VR, nonimmersive VR	No	None
Rajendram et al. (2022)^53^	No	None	None	Unspecified VR	No	None
Saliba et al. (2022)^54^	No	Minimal	None	Immersive VR only	No	Additional Characterization
Savaş et al. (2023)^55^	No	Minimal	None	Unspecified VR	No	None
Singleton et al. (2023)^56^	No	Substantial	Minimal	Immersive VR, nonimmersive VR	N/A	Immersivity
Smith et al. (2022)^57^	No	Minimal	Minimal	Immersive VR, nonimmersive VR	No	Interactivity
Su et al. (2023)^58^	No	None	None	Immersive VR, nonimmersive VR, AR + MR	No	None
Tas et al. (2022)^59^	No	None	Substantial	Immersive VR only	No	Interactivity
Vassantachart et al. (2022)^60^	No	Substantial	Minimal	Immersive VR, nonimmersive VR	N/A	Immersivity
Vitagliano et al. (2023)^61^	No	Substantial	Substantial	Immersive VR only	Interactivity level	Interactivity
Wang et al. (2022)^62^	No	None	None	Immersive VR only	No	None
Wong et al. (2022)^63^	No	Minimal	Substantial	Immersive VR, nonimmersive VR	N/A	Immersivity
Ye et al. (2023)^64^	No	Minimal	Minimal	Immersive VR, nonimmersive VR	No	Immersivity, Interactivity

### Quality of hardware description

In general, hardware was almost universally described, as it was generally relevant to synthesis scope or inclusion criteria. Looking more specifically at whether hardware details were reported for included studies, 18 (30%) did not include any details about specific hardware configurations for individual trials, while 15 (25%) included minimal descriptors (e.g., “VR glasses,” “head-mounted displays”^41^) and 28 (46%) included substantial descriptors (e.g., eMagin z800 3DVisor, Oculus Rift, Google Daydream^59^).

Across all papers, analysis of hardware descriptions included for the individual studies revealed that a minority (*n* = 19, 31%) included only immersive VR equipment, most typically defined by use of a head-mounted display. This was often predetermined in synthesis search and/or inclusion criteria but was occasionally incidental. Almost half (*n* = 29, 46%) included both immersive and nonimmersive VR, including four (7%) studies that incorporated augmented reality or mixed reality in addition to VR. This included reviews that clearly specified exclusive inclusion of “immersive VR” in their methods section or reported included studies as “immersive” despite containing interventions that would more typically be considered nonimmersive. For example, Lier et al.^42^ define immersive VR as “any intervention using a head-mounted device that completely blocks the view of the users’ real-world surroundings” and specifies that nonimmersive VR was excluded. However, some included studies use less immersive or nonimmersive interventions, such as watching cartoons with 3D glasses. Similarly, while Garrido-Ardila et al.^29^ did not require exclusively immersive VR in their methods, their results reported that all included studies were “immersive” even though the review included several nonimmersive interventions according to our definition (e.g., Wii exercises, Xbox Kinect). Two (3%) reviews included only nonimmersive VR-focused studies; in both cases this was incidental to the nature of studies meeting inclusion criteria and was not predefined. Several reviews (*N* = 11, 18%) did not specify VR or XR type in either their methods or when describing included studies in their results.

### Examination of intervention factors

Beyond descriptions of individual studies, reviews often discussed specific intervention elements and the influence of these elements on study outcomes. This ranged from broad statements about the “types” of studies captured in the review (e.g., immersive, distraction-based) to statistical analysis of intervention efficacy stratified by intervention factors. Over a third of the papers (*N* = 22, 36%) discussed the role of immersion in XR interventions in some capacity, while fewer (*N* = 12, 20%) discussed the role of active or interactive interventions as opposed to passive ones. Additional characterization was seen in 16 papers (26%), including commentary on aspects such as gamification, whether software was commercial versus custom-made for the study, and the 2D versus 3D nature of experiences. Statistical evaluation of intervention efficacy based on content or hardware factors was somewhat limited. Among the set of 39 papers that included a meta-analysis (wherein moderators could be statistically assessed), 10 (26%) explicitly evaluated the role of intervention elements in intervention efficacy. Factors considered in these analyses included: content type (*N* = 3), interactivity (*N* = 3), immersive versus nonimmersive approaches (*N* = 2), interventions as adjunctive versus standalone (*N* = 2), and patient age group targeted by the intervention (*N* = 4). Twenty-nine papers with meta-analyses (74%) did not explicitly evaluate the impact of intervention factors on efficacy.

## Discussion

Through formal quantitative content analysis, this study determined that the way MXR interventions are defined and characterized in the published evidence synthesis literature varies widely across several dimensions. The body of reviews and meta-analyses published in the domain of MXR interventions for pain management was highly heterogeneous in terms of how key features of specific XR interventions are characterized and synthesized. This included a full 30% of publications that contained no information about the content of synthesized XR interventions, and 30% that did not provide information about the XR hardware used to deliver interventions. These findings are consistent with the general conclusions drawn by Vinderman and colleagues^4^ who performed an umbrella review of evidence synthesis in VR and pain management. This umbrella review suggested that many synthesis publications did not describe included studies with adequate detail and that sources of heterogeneity between studies were rarely discussed. These findings suggest a need for change in reporting approaches. Inclusion of intervention information is critical, as it defines the universe of MXR interventions to which synthesis results can be generalized.

Descriptions of the overall scope of reviewed papers and inclusion criteria for reviewed studies can, at times, provide relevant information about both content and hardware included in synthesis. However, descriptions of these parameters are rarely fine-grained enough to provide a clear vision of the interventions comprising synthesis. This is because MXR pain interventions are complex, multicomponent, and very heterogeneous. For example, Lier et al.^42^ evaluated 122 randomized controlled trials for a variety of pain types. Interventions varied from watching a 2D video to playing a 3D immersive VR game, and content descriptions were wide-ranging as well, including cartoon videos, hypnotherapy, meditation, rollercoaster simulations, and patient education. It therefore becomes a necessity to provide intervention details for each reviewed study.

Relatedly, the wide variation in platforms that were designated as VR or XR in reviewed studies also underscores a need to provide detail about hardware platforms employed in reviewed studies. At times, we found contradictory information about intervention hardware platforms that synthesis scope was described as including. For example, some claimed inclusion of only immersive VR systems, while the body of reviewed studies contained interventions that would more typically be defined as nonimmersive. It is possible that some synthesis authors may have assumed that interventions described as VR in original study papers were immersive in nature. In addition, the normative definition and characteristics of VR can vary over time, between disciplines, or along other lines yet to be explored,^65^ resulting in such discrepancies.

The current study offers several important observations in considering whether and to what extent this body of recent research syntheses answers key questions about MXR efficacy in various pain management areas. First, it is notable that 61 evidence syntheses, all focused on pain, with a median of 10 reviewed studies each were published in a 3-year span. This suggests a robust primary literature as well as a research community that is very engaged in the synthesis process. Many primary studies were likely included across multiple syntheses; however, several were relatively narrow and nonoverlapping in scope (e.g., women with fibromyalgia,^20^ phantom limb pain in patients with limb amputations,^27^ venipuncture in children^46^) suggesting a limit to the amount of shared source material.

We also observed that many syntheses that did characterize content and hardware included primary studies that relied on approaches that are now somewhat dated and may have limited correspondence to contemporary MXR approaches. This includes modalities such as phone-based headsets and legacy video game consoles. To the extent that early intervention studies rely on similar mechanisms of action to today’s tools, they can provide useful aggregate information about general effect robustness. However, if the majority of interventions reviewed in a given synthesis are based on such dated hardware and software, this severely limits generalizability to contemporary MXR interventions. Likewise, synthesis of solely relaxation and distraction-based XR pain intervention studies does not provide needed information about the likely efficacy of XR pain interventions using other mechanisms, such as mindfulness or education. Such limitations only become evident when primary study inclusion criteria are clear and included studies are well-characterized.

Given tradeoffs between including a wider versus narrower scope of MXR interventions, a combined approach may be particularly useful for synthesis. We find this in the form of publications that use of a broader set of inclusion criteria to capture a larger swath of relevant studies, alongside several sub-group or moderator analyses that provide targeted assessment of specific content, hardware, study design, and other features in evaluating intervention efficacy. Our analysis suggests that the importance of such features is recognized in the MXR research community; intervention characteristics like level of immersion and interactivity were frequently discussed at least conceptually in our sample of synthesis publications. Despite this, only a quarter of reviews that contained meta-analyses also assessed the role of specific intervention features statistically. Engaging in this analysis was fruitful and can be used to guide recommendations and best practices for developing more effective MXR interventions in the future. For example, Norouzkhani and colleagues^49^ concluded that immersive XR interventions were effective for pain reduction during burn wound care whereas nonimmersive XR interventions were no different from control. Perhaps unintuitively, Lier and colleagues^42^ found that XR interventions for pain, broadly, may be more effective when they are not interactive. It is likely that MXR intervention features function differently in different contexts. For example, while Norouzkhani^49^ found immersive XR interventions to be superior for burn wound care, this pattern may not hold for other pain conditions, in other populations, or using other outcome measures. More research evaluating the interaction of these features is certainly warranted. To get there, however, it is critical to start uniformly characterizing primary study interventions and their features. Such detailed characterization was relatively rare in the studied papers, with only 23% including substantial content descriptions and 46% providing substantial hardware descriptions.

Based on current findings, we make several basic recommendations to guide future evidence syntheses in the MXR domain. These are listed in Table 4.

**Table 4.** Recommendations to Guide Future Evidence Syntheses in Medical Extended Reality

1	Carefully consider the question or claim being addressed by the evidence synthesis in order to carefully match this conceptual aspect with the scope of included primary studies and the intervention details being extracted and explored.
2	Define MXR interventions holistically, including content and hardware features at a minimum, and provide characterizations of interventions that include key dimensions such as immersivity, interactivity, and proposed mechanism of action where possible.
3	Begin with a clear definition of XR that is explicitly stated and used as a criterion for study inclusion. Consider that XR technologies have had many definitions, and not all primary studies that describe their intervention as XR will meet modern definitions of the technology.
4	Meta-analysis is not always possible, such as in cases where the relevant literature is too small or heterogeneous. However, consider conducting meta-analysis whenever possible to better characterize the variability and effect size of study outcomes.
5	When possible, explicitly assess the influence of key factors in moderation or sub-group analyses. This can greatly expand potential conclusions and thus heighten synthesis impact.

MXR, medical extended reality.

### Limitations

This analysis has several limitations. First, its scope was limited to a 3-year period which represents recent synthesis projects in the field but is also only one snapshot in time. Trends and best practices in the field will continue to change over time, and approaches to reporting in reviews and meta-analyses will change with them. Representation of commercially available pain interventions (which may be automatically tied with particular content and hardware configurations) was very limited in the publications reviewed here. We also coded only a limited number of features associated with each synthesis publication, as we limited the scope of analysis to address only the current research questions. For example, aspects such as intervention efficacy, overlaps in reviewed studies, and time frame of studies included in syntheses were beyond the scope of our study analyses. Study quality was also beyond scope but is clearly a critical element in conclusions about MXR efficacy for pain management. Finally, we included only synthesis publications related to pain in the current analysis; the extent to which the patterns reported here generalize to other MXR use cases is not known.

## Conclusion

Through enhanced reporting and characterization of intervention elements, the MXR synthesis literature will become more sophisticated and better allow the research and practice communities to assess gaps in the evidence base. This is a critical step in the research process, as it enables evidence-based judgements of the potential for MXR intervention efficacy, which approaches are most effective in which cases, and predictions about which future intervention directions will result in the most health benefit for the population.

## Abbreviations Used

AR: augmented reality
MXR: medical extended reality
VR: virtual reality
XR: extended reality
